# Relationship between the Size and Inner Structure of Particles of Virgin and Re-Used MS1 Maraging Steel Powder for Additive Manufacturing

**DOI:** 10.3390/ma13040956

**Published:** 2020-02-20

**Authors:** Kateřina Opatová, Ivana Zetková, Ludmila Kučerová

**Affiliations:** Regional Technological Institute, University of West Bohemia, 30100 Pilsen, Czech Republic; zetkova@rti.zcu.cz (I.Z.); skal@rti.zcu.cz (L.K.)

**Keywords:** additive manufacturing, FIB, EBSD, EDS, maraging steel

## Abstract

Additive manufacturing (AM) is today in the main focus—and not only in commercial production. Products with complex geometry can be built using various AM techniques, which include laser sintering of metal powder. Although the technique has been known for a quite long time, the impact of the morphology of individual powder particles on the process has not yet been adequately documented. This article presents a detailed microscopic analysis of virgin and reused powder particles of MS1 maraging steel. The metallographic observation was performed using a scanning electron microscope (SEM). The particle size of the individual powder particles was measured in the SEM and the particle surface morphology and its change in the reused powder were observed. Individual particles were analyzed in detail using an SEM with a focused ion beam (FIB) milling capability. The powder particles were gradually cut off in thin layers so that their internal structure, chemical element distribution, possible internal defects, and shape could be monitored. Elemental distribution and phase distribution were analyzed using EDS and EBSD, respectively. Our findings lead to a better understanding and prediction of defects in additive-manufactured products. This could be helpful not just in the AM field, but in any metal powder-based processes, such as metal injection molding, powder metallurgy, spray deposition processes, and others.

## 1. Introduction

Additive manufacturing technology (AM) is able to create high-quality intricately shaped metal parts for use in the aerospace, automotive or medical industries. Unlike conventional subtractive manufacturing processes, where the material is removed in order to obtain the desired shape, many additive manufacturing techniques work on the principle of adding and sintering individual layers of material. In this way, it is possible to produce complex-shaped parts that may contain a plurality of internal elements, such as cooling channels [1,2]. Only a minimum amount of material is scrapped during the production because the powder used for AM can be recycled and reused. The powder that remains in the build chamber after the part has been produced is collected manually and sieved. The size of the sieve openings depends on the requirements of the AM technology and on the desired quality of the parts to be built. This makes it possible to efficiently use up to 97% of the input material [3,4].

To obtain builds with the desired properties, the powder should be of high quality and its properties should be consistent and well-defined across all batches. The size distribution of the powder particles profoundly affects product quality. The product quality also depends on whether the input material is new or re-used powder. All batch-specific defects in the powder need to be identified. These may include the presence of non-spherical particles, crushed particles, inclusions or gas entrapped in the particles during their production, particles of inappropriate size, or undesirable particle size distribution in the batch. The occurrence of defects depends on the methods of powder production, handling, recycling and preparation for the AM process [3,4,5,6].

Metal powders are typically manufactured using either mechanical or chemical processes. The first group includes water atomization, mechanical alloying, electrolysis, and the latter involves oxide reduction and various chemical methods. The choice of the production process depends on the requirements placed on the powder, such as physical and chemical properties, and the batch size. Chemical and electrolytic methods produce high-quality materials. Mechanical preparation, such as milling, is good for preparing high-hardness or oxide-based materials. Most powders used in AM are produced by atomization or milling. Both are extremely energy-consuming processes [7,8,9].

Atomization is probably the most widely used method, offering the broadest scope of use. In the process, molten metal is broken up and carried by a high-speed inert gas (nitrogen, argon, or helium) through a disperser, which produces droplets that cool rapidly. The resulting spherical particles are collected in a container. Their cooling rates range from 102 to 107 °C·s^−1^. Rapid solidification (RS) takes place, leading to particles with a typical size of up to 150 μm, although larger sizes are not unusual. Ideally, the particles obtained by gas atomization should be smooth and spherical. In practice, some include ‘satellites’: smaller particles that adhere to larger ones. The likely cause for this is that smaller particles swirl in the collecting container and collide with larger, still partially melted particles that have just entered the container [3,5,7,8,9].

Quality control of metal powders consists of either sieve analysis according to ASTM B214 or, as in this experiment, laser diffraction analysis [10]. The quality control process is significantly impaired by particles with irregular shapes, be they satellites or non-spherical particles.

Sieve analysis—as described in ASTM B214 standard—separates metal powder particles based on their size. Dry sieves with various sizes of openings from 5 to 850 μm are used. The method provides very good repeatability but no information about the shapes of individual particles. However, knowing the powder particle size distribution is essential for delivering high-quality AM products. Sieve analysis provides percentages of volume fractions of particles in certain size intervals [11].

The principle of the method consists in stacking sieves with increasing mesh sizes on top of each other. The stack, with a powder sample placed on the top sieve, is then shaken (mostly by mechanical means) until the residues on each sieve contain only those particles that have fallen through the above sieves but cannot pass through the underlying one. This procedure is too time-consuming for commercial environments. It is usually performed either during changeovers or periodically at certain time intervals [7].

In contrast, laser diffraction analysis allows for real-time monitoring of particle size distribution during production and, at the same time, provides instant feedback for optimizing the production process [3].

It is one of the most widely used methods of measuring particle size. It uses the principle of coherent light scattering. Particles suspended in a slurry pass in front of a laser beam source. The particle size distribution is determined from the angle and intensity of the diffracted light. Today, laser diffraction can effectively measure particles within a size range of 0.01 μm to 5000 μm, which includes the entire size interval required for AM powder production. The output from this method is the same as that from powder sieve analysis: volume fractions of particles in certain size intervals [7].

This method cannot be used for identifying the shapes of particles, yet the shape criterion is important for ensuring that metal powder flows continuously during the AM process. Determining the circularity of individual particles is time-consuming, and is usually done manually with the aid of a microscope [3].

## 2. Materials and Methods 

The EOS Maraging Steel MS1 metal powder investigated in this study was developed by the company EOS (Krailling, Germany) specifically for use with their EOSINT M additive manufacturing systems. These are “3D printers” for metals, equipped with a 400 W laser source with a wavelength of 1060–1100 nm. The maximum available build height/*z*-axis stroke of these machines is 325 mm, which includes the building platform.

The powder is produced by atomization. Its chemical composition corresponds to the US grade 18% Ni Maraging 300, the European 1.2709 and the German X3NiCoMoTi 18-9-5 grade steels (Table 1). This steel is characterized by very good mechanical properties and heat treatability after the AM process. Its heat treatment sequence consists of stress-relieving at 820 °C for 1 h and age hardening at 490 °C for 6 h with cooling in furnace, which leads to high strength and hardness in the final product [12].

The particle size range reported by the manufacturer is from 10 µm to 63 µm, the mean size being 50 µm. In this study, the actual particle size distribution was examined. To identify differences between new (virgin) and used powders, samples were observed in a scanning electron microscope (SEM). All the above-mentioned defects were examined, as they adversely affect the quality of the resulting product (undesirable particle size distribution, out-of-roundness and internal defects in individual powder particles) [13]. The particles were measured in a Zeiss AURIGA scanning electron microscope (Oberkochen, Germany) equipped with a field emission gun of the Schottky type with an electron beam resolution of 1 nm. The system also featured a Focused Ion Beam (FIB) gun, detectors of secondary and back-scattered electrons (SE, BSE), energy-dispersive X-ray spectroscopy (EDS) detector, electron backscatter diffraction (EBSD) detector, and also provided scanning transmission electron microscopy (STEM) capabilities for thin specimens.

Three types of samples were prepared:

- virgin powder—taken from randomly chosen locations in a newly opened barrel of powder
- reused powder—powder which had passed through a recycling sieve (the mesh size was 80 μm; alternatively, 63 μm can be used)
- residual oversize powder from the sieve after recycling.

All the specimens were mounted on SEM stubs on carbon adhesive discs. The diameters of the metal powder particles were measured using SmartTiffV3 software (version V03.00) from Zeiss and ImageJ software (ImageJ/Fiji 1.46) [14].

**Figure 1:**
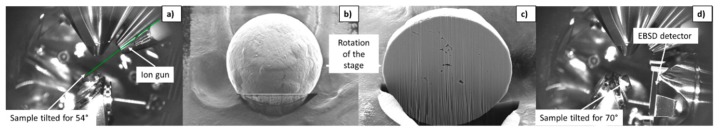
Particle being prepared for EDS and EBSD analyses. (**a**) Ion milling and polishing, (**b**) FIB image during milling, (**c**) a sample in a position for EDS analysis, (**d**) a sample in a position for EBSD analysis.

The size distribution and circularity of particles in each powder sample were measured. The surfaces of individual powder particles were examined. The surfaces of the reused powder particles differ from those of the virgin powder particles, probably because of differences in the process of formation of some of them. Several particles were cut using FIB. The milling sequence involved progressive removal of several dozen slices with an approximate thickness of 200 nm and observation of the particle cross-section. The purpose was to examine the inner structure and spatial distribution of defects in the particles, such as inclusions and chemical inhomogeneity. Approximately 30 virgin powder particles and 30 reused powder particles were sliced in this manner. Representative particles from several size categories were chosen in order to gain comprehensive knowledge about the dependence of their inner structure on their size and manufacturing history. The parameters used for milling were: a current of 100 nA for coarse milling, 30 nA for coarse polishing and 10–3 nA for fine polishing. With small particles of virgin powder, lower current levels had to be used for final polishing for microstructure analysis. The cut surfaces were characterized using EDS and EBSD. A special holder for the SEM stubs was used for this purpose. The holder was pre-tilted to 54°, making it possible to cut the sample from one side and then carry out EDS and EBSD analyses (Figure 1).

**Figure 2:**
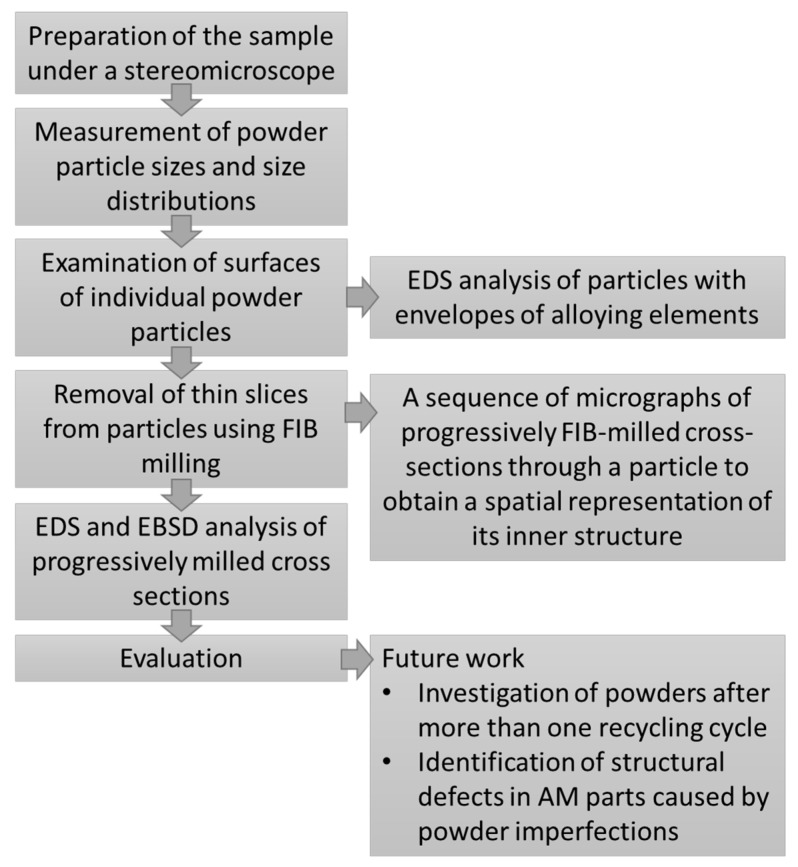
Flow-chart of preparation and examination of powder samples.

In this paper, band contrast maps and inverse pole figure (IPF) maps obtained using EBSD are presented. Band contrast (BC) is an electron backscatter pattern (EBSP) quality factor derived from the Hough transform that describes the average intensity of the Kikuchi bands with respect to the overall intensity within the EBSP. BC maps show the microstructure in a qualitative manner. Because EBSPs along grain boundaries tend to show poor band contrast, they appear dark on the map. Grain boundaries can thus be identified in the undeformed structure. IPF orientation maps show the size, shape, and orientation of grains in the powder particles. Each individual crystal orientation is colored differently. The color coding for orientations is presented in a Standard Stereographic Triangle (SST), which is inserted in IPF orientation maps [15,16,17]. A complete flow-chart of steps to determine the properties of the metal powder and defects in its particles is shown in Figure 2.

## 3. Results

### 3.1. Virgin Powder

**Figure 3:**
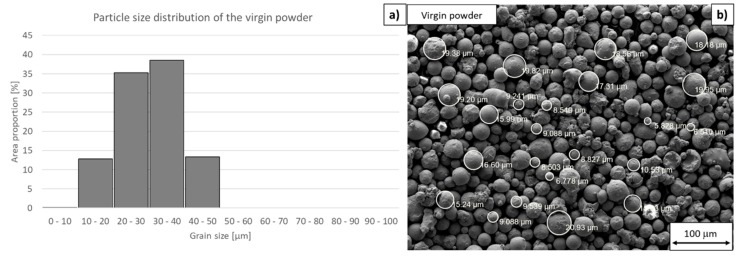
Virgin powder. (**a**) Particle size distribution (using the ImageJ software, 130 particles were measured for statistical significance), (**b**) tentative particle size measurement in a scanning electron micrograph using the SmartTiff software.

The particle size of the virgin powder was measured using an electron microscope. It was found that the proportion of small particles (<10 µm) was larger than that declared by the manufacturer (approximately < 10 wt.% of the batch) (Figure 3). The mean particle size was a mere 25 µm. The mean circularity was found to be 0.93 (unity would denote a perfectly circular particle). The measurements carried out in this study might differ slightly from those made by the manufacturer because of the measuring method, which was carried out by SEM in this paper and according to ASTM B214 standard by the manufacturer. Despite that, the fact that the fraction of particles with small diameters in the sample was larger than declared is undeniable.

After their diameters had been measured, some particles were cut using a focused ion beam. Representative particles from each size category were randomly chosen for this examination. 

**Figure 4:**
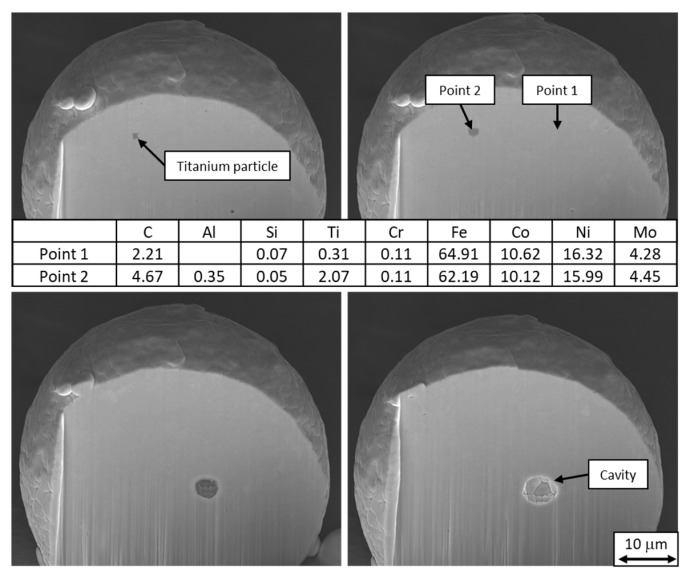
Micrographs of a progressively FIB-milled (from top left to bottom right) virgin powder particle containing a sharp-edged titanium inclusion and a cavity and chemical composition in weight percent measured by EDS. Different shapes of these defects were revealed in successive sections.

**Figure 5:**
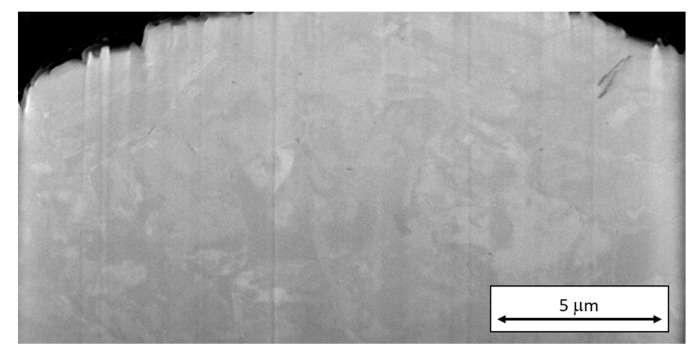
A crack below the surface of a virgin powder particle in a scanning electron micrograph taken during FIB milling. The inner structure of the particle has already been partially revealed by etching with the ion beam.

Micrographs taken using secondary electron imaging showed that some defects were present mainly in particles with larger diameters. These were titanium segregations and cavities. To better understand and visualize the defects, EDS and EBSD analyses were performed on the cross-sections through the particles with an emphasis on the shapes of the defects and on the differences between the large and small particles. All the cavities in the virgin powder were spherical, with diameters up to 10 µm. Small cracks were found, the size of which did not exceed 5 µm. In most cases, they were just below the surface (Figure 4 and Figure 5).

**Figure 6:**
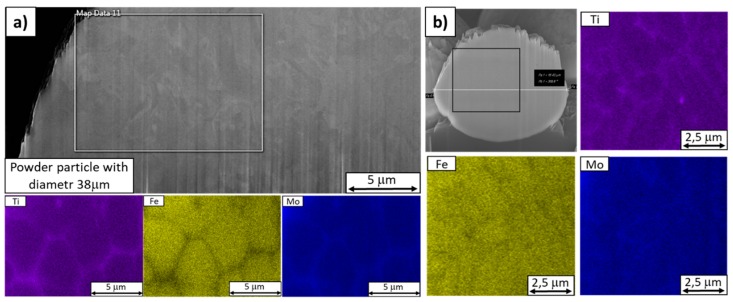
EDS maps of virgin powder particles. (**a**) A larger particle with a diameter of 38 µm with a partially etched microstructure, (**b**) a smaller particle, 16.5 µm in diameter.

**Figure 7:**
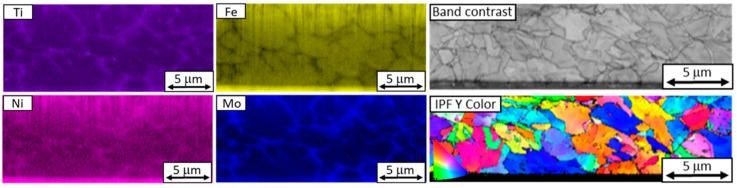
EDS and EBSD images of a section through a virgin powder particle with chemical segregation along the boundaries of its structural units. A band contrast map and an IPF map reveal the grain structure of the particle.

EDS analysis of the virgin powder identified chemical segregation in its particles. It was more severe in particles with larger diameters (Figure 6a). The most significant cases of chemical heterogeneity involved titanium, iron, and molybdenum (Figure 6a,b). Segregated titanium and molybdenum formed closed units in particles of every size. In larger particles, the internal structure was visible in the secondary-electron images of the FIB-milled surfaces due to slight ion etching (Figure 7). This internal structure did not fully correspond with the elemental maps. Nevertheless, the boundaries of the structural units were sometimes delineated by segregated molybdenum.

EBSD analysis revealed the same internal structure pattern as the one which had been partially revealed by ion etching and imaged using secondary electrons. It was found that segregated titanium and molybdenum occupied the boundaries of the units of this inner structure. The size of these units in the virgin powder was from several hundred nanometers to several micrometers, as seen in the band contrast map in Figure 7.

### 3.2. Reused Powder

Newly formed particles account for only a small fraction of the volume of the reused powder sample. There are differences between these newly formed particles and the original particles from the virgin powder in terms of surface appearance, size, circularity, and inner defects. The newly formed particles can be several times larger than the virgin powder particles, as they cool more slowly when they form. These newly formed particles were the focus of the analysis of the reused powder.

**Figure 8:**
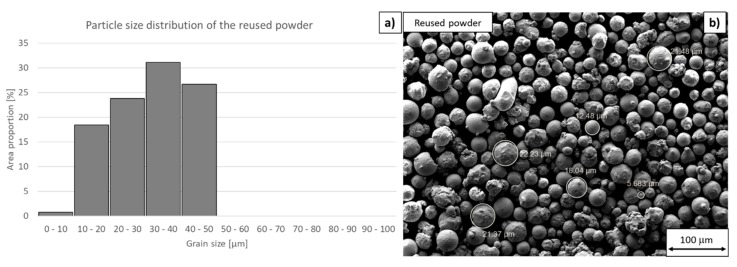
Reused powder. (**a**) Particle size distribution (using ImageJ software, 130 particles were measured for statistical significance), (**b**) tentative particle size measurement in SE micrograph using SmartTiff software.

Measurements of particle size distribution and circularity indicated a much larger proportion of small particles (diameter < 10 µm) in the reused powder than in the virgin powder. This is probably due to the difference between the conditions for forming new particles during the building process and during atomization. The mean size of the reused powder particles was only 23 µm. The mean circularity was 0.91 (Figure 8). Although the mean size of the reused powder particles was not much different from that of virgin powder, the difference between the particle size distributions in these powders was significant. There were more particles with small diameters (<10 µm) and with large diameters (≅50 µm) in the reused powder than in the virgin powder. The number of mean-size particles was much lower than in the virgin powder.

**Figure 9:**
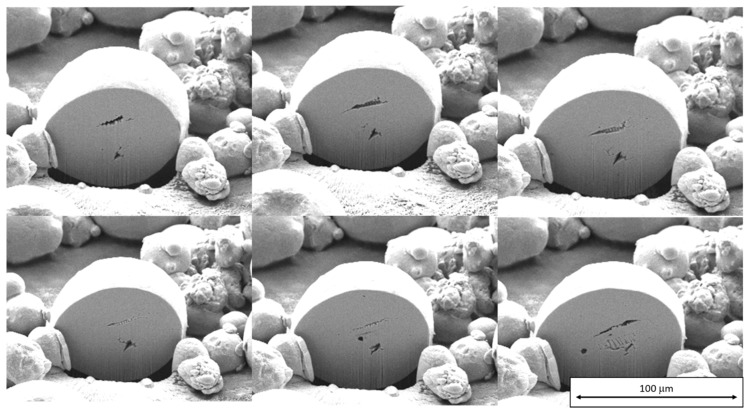
Scanning electron micrographs taken during FIB milling of a reused powder particle. Successive sections through defects in the particle can be seen. The shapes of these defects in the reused powder are not strictly spherical, in contrast to those in the virgin powder.

Reused powder particles were more often distorted and non-round than the virgin powder particles. The cavities in them were not strictly spherical, unlike those in the virgin powder. Instead, they had various elongated shapes, as revealed by FIB milling (Figure 9).

**Figure 10:**
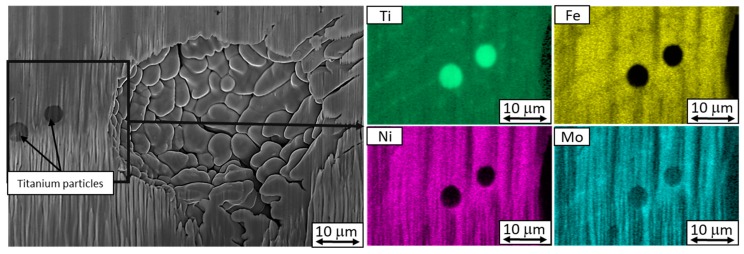
Scanning electron micrograph of an inclusion and titanium particles in a reused powder particle. Cracks can be seen along the boundaries of dendrites. Titanium particles in reused powder are more circular than those in the virgin powder.

It was found that all reused powder particles of about 40 µm or more in size were certain to contain cavities. Cavities can also occur in smaller particles. Reused powder particles showed significant segregation of titanium. In contrast to virgin powder, reused powder particles contained globular titanium particles (Figure 10).

**Figure 11:**
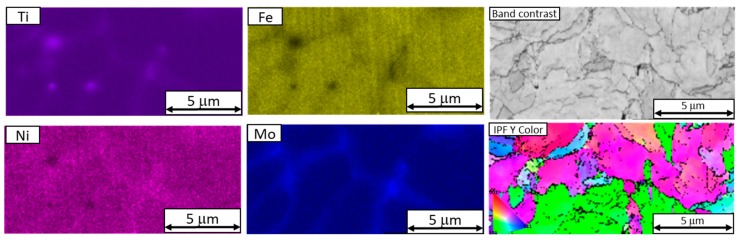
EDS and EBSD maps of a reused powder particle showing chemical segregation along the boundaries of the cellular structure. The band contrast map and the IPF map on the right show the grain structure.

The internal structure of the reused powder particles consists of units delineated primarily by molybdenum, and also by titanium and nickel. These units are larger than those in the virgin powder (up to 10 µm), as shown in the band contrast map (Figure 11).

Chemical heterogeneity was observed in the surfaces of those particles which had been newly formed during the AM process. It was probably caused by slower cooling of these particles upon formation, which was also reflected in their different surface morphology. Two types of layers were found on their surface:

- Titanium layers;
- Layers of titanium, molybdenum, and nickel.

These layers can cause defects in AM products because they are difficult to melt. They form because of the different densities of their constituting elements. The density of titanium is almost two times lower than that of the other elements. On the other hand, the melting point of titanium is much higher than that of the experimental material itself. This means that the experimental material can melt more readily while in its initial condition—before its chemical constituents have migrated to form the layers on the newly formed particles.

**Figure 12:**
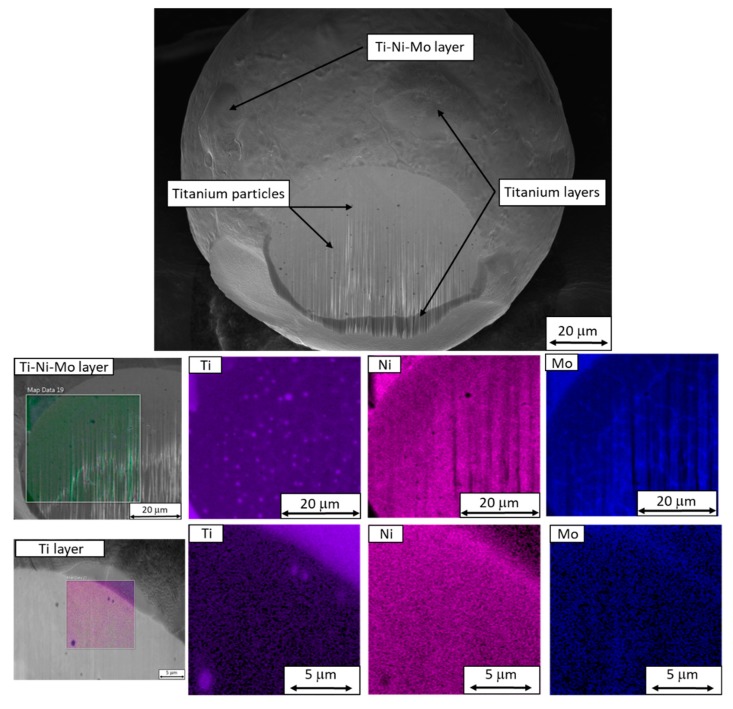
EDS maps of a reused powder particle showing the differences between a Ti-layer and a Ti-Ni-Mo layer. Titanium particles and molybdenum segregation in the cellular structure can be seen.

In both reused and virgin powders, there was significant chemical segregation in their structures, namely with respect to titanium, molybdenum, and nickel. In both powder samples, the boundaries of microstructural units were delineated by molybdenum. Their size ranged from hundreds of nanometers to several micrometers. In the reused powder, they were larger than 5 µm, and therefore larger than in the virgin powder, where they were smaller than 5 µm (Figure 12).

### 3.3. Residual Oversize Powder from the Sieve

Only particle size distribution and circularity were measured on this powder sample, as this powder would not be used for further additive manufacturing. The size distribution showed a drop in the number of particles between 20 µm and 60 µm, as most of them had fallen through the sieve and been collected for reuse.

**Figure 13:**
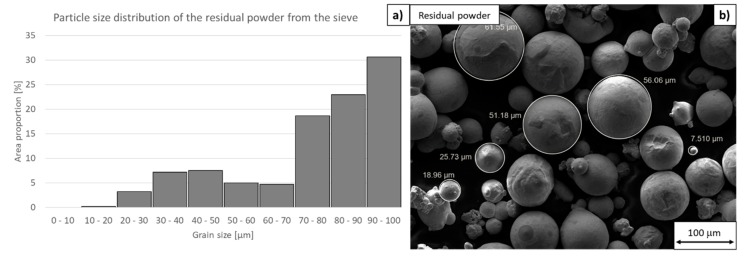
Residual oversize powder from the sieve. (**a**) Particle size distribution (using ImageJ software, 130 particles were measured for statistical significance), (**b**) tentative particle size measurement in an SEM image using SmartTiff software.

The mean particle size was 58 µm. The mean circularity was as low as 0.88. Many satellites and particles with surface layers of segregated titanium, nickel, and molybdenum were found in the sample (Figure 13). The motivation for measuring a sample of residual oversize powder from the recycling sieve was to observe a larger quantity of particles with imperfections. This sample consists almost exclusively of particles with layers of segregated alloying elements and particles with defects. Yet, this fact does not prove that most of the imperfect particles are eliminated during powder recycling. Examination of this powder sample gives an opportunity for a detailed study of the defects in the powder particles which were newly created during the AM process.

## 4. Discussion

Given that the production of AM metal powders will be worth more than $500 million in 2019 and as much as $900 million in 2023 (40% annual growth), it is essential to be able to characterize the properties of these powders. The ultimate goal is to predict the quality of AM products and achieve savings in their production. Once the dependence of the behavior of metal powders on their particle shape, defects and size distribution is known, powder production can be optimized to obtain defect-free products [4,18].

In this study, measurements of particle size distribution revealed a major discrepancy between the mean particle size declared by the supplier (50 µm) and the measured mean size (25 µm). Furthermore, the particle size distribution in individual samples and their circularity were investigated (Table 2). These parameters were significantly different for the virgin and reused powder. The change in the particle size distribution of the reused sample was due to the formation of new powder particles during the printing process itself, where the molten metal particles were blasted by a laser beam and highly heterogeneous particles whose size and shape could not be predicted were produced. The share of powder particles with internal defects in the form of cavities increased proportionally to particle size. Newly formed particles contained more cavities of this kind, possibly due to their formation mechanism being different from gas atomization [5]. By examining sufficient numbers of powder particles (approximately 30 virgin powder particles and 30 reused powder particles), it was found that no cavities were present in particles with diameters smaller than 30 μm. The presence of cavities in the individual powder particles can be explained by slower cooling rate during the formation of larger particles and the closure of the gas used during the atomization or printing process in individual particles. Moreover, during printing, the formation of new particles is not as dynamic as in the atomization process, which led to the formation of a bigger amount of large diameter particles that tend to have more internal cavities.

**Figure 14:**
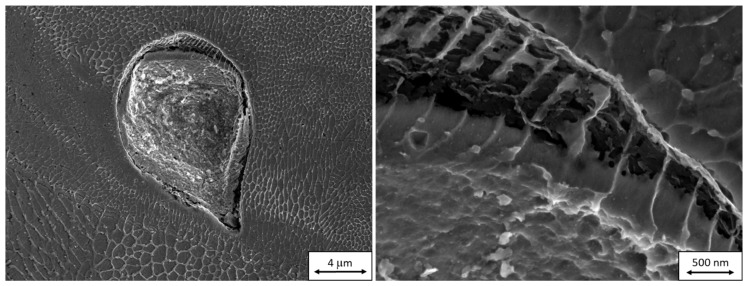
Defect in as-built material, possibly caused by a particle that failed to melt during AM.

Other defects that were observed on the newly formed powder particles were chemical segregations. The alloying elements, in particular Ti, Mo and Ni, formed envelopes on the surface of the particles. Their formation can be explained by the fact that the process of formation of new particles in the chamber of 3D printer is less dynamic than the formation of new powder particles during atomization. For this reason, the elements have time to segregate and the resulting particle has an inhomogeneous chemical composition. This heterogeneity is greater the larger the radius of the particle is. Thus, particles with a radius of about 50 µm usually exhibit both types of defects, internal cavity and chemical segregation on the surface. Chemical segregation on the surfaces of some reused particles can cause significant problems during their use in AM. Titanium exhibits the strongest tendency to form coatings that enveloped newly formed particles [19]. Its density is almost two times lower than that of the other chemical elements in this material. Particles with such coatings were therefore likely to accumulate in certain locations during handling with the powder. As a result, they would be processed together at a single point during the building process. For instance, if a batch of metal powder is shaken during refilling of the build chamber, lighter particles containing more titanium will float to the top of the powder bed. Their higher melting point can then lead to defects in the final AM part (Figure 14). The formation of the segregation layers will be the subject of further investigation because, despite the small amount of titanium in this material (less than 1 wt.%), there is a tendency for titanium layers to form. Moreover, the amount of titanium in the segregated layers or particles is more than two times higher than in the rest of the experimental material (more than 2 wt.%).

The relationship between EDS and EBSD data and particle structure will be investigated in detail in future work. Segregations of molybdenum were found to correspond with dendrite boundaries. EBSD band contrast maps revealed the martensitic structure of the material. This shows that the microstructure of the powder particles is similar to that of the AM product, in which dendrite boundaries and the substructure within the martensite can be revealed by various etching techniques. Dendrites were formed either during gas atomization or during formation of the new particles in the chamber of 3D printer. They are due to the high cooling rates during both processes [20,21,22,23]. The microstructure of newly formed particles tends to be coarser than in the particles of virgin powder. This is caused by the slower cooling rate during forming of these particles. Their forming process was less dynamic than atomization which led to segregation of alloying elements that were able to form envelopes on the surface of new powder particles. The grain size of particles formed in the chamber of the printer will not change, but the envelopes on particle surface will affect the melting process of such particles during printing. Newly formed particles grain sizes could be influenced by the laser beam parameters used for printing, as stated by Ali et al. [24]. Their grain size could be both smaller and bigger with respect to the laser parameters. With lower velocity, larger particles with more pronounced chemical heterogeneity and coarser grains would be produced, as the chemical elements would have time to segregate and the grains will grow.

It is clear that gradually changing properties of the powder particles during multiple recycling is connected to the properties of the printed parts. Nowadays there is no sufficient standardization on feedstock powders for AM. The change in chemical composition and segregation of the alloying elements on the surface of the particles will affect the printing process and the properties of printed part as it was stated by Thomas et al. [5].

## 5. Conclusions

In this study, imperfections that occur in powder particles used for AM, metal injection molding, powder metallurgy, spray deposition, and other processes that use metal powder as the input material were identified. Our findings can lead to a better understanding of defects in the final products that these technologies produce. The investigation included a study of virgin powder and a study of reused powder. Both types of powder material were observed by SEM, where their size was first measured. The results of virgin powder were compared with the declared particle size distribution of the metal powder from the manufacturer. These results varied considerably, and it was found that a large number of small powder particles were present in the material. The difference in particle size distribution may be due to different measurement methods, but a large number of small particles are irreversible. Size measurement of the reused powder has shown that new particles of much larger diameter are formed during the additive manufacturing process. A significant difference was also observed in the surface morphology of the new and reused powder. Envelopes made of alloying elements with a high titanium content are present on the surface of the particles of the reused powder. The next step was to gradually cut thin layers of material from individual powder particles using an ion beam. Internal defects such as inhomogeneous chemical distribution or cavities have been observed. The distribution of the chemical elements of the virgin powder was mostly homogeneous with little occurrence of titanium particles. In contrast, large amounts of titanium particles and segregated molybdenum were present in the reused powder particles. The occurrence of these internal defects affects the quality of the final product mainly due to different melting temperature of the envelopes of alloy elements which are present on the surface of the newly formed powder particles. These envelopes may not melt properly during the next printing process and remain in the printed part as chemical heterogeneities and potential defects origins. The cavities within the individual particles do not present such a problem for the resulting product, provided that the same inert gas is used at all times. If so, these particles will melt during the next printing process, the cavities will disappear, and the protective atmosphere will not be disturbed. EBSD analysis revealed that chemical elements replicate the cell structure within the particles, but at the same time the particles have a grain structure. By gradually cutting off the material layers, it was also possible to observe the internal cavities in the particles and their shape. Particles of smaller diameters of the virgin powder are mostly free of such defects, but larger particles have spherical cavities. In the reused powder, the presence of cavities in the particles is more frequent, the voids also occur in smaller particles and are not strictly spherical in shape. Repeated use of the powder increases the possibility of internal defects in the resulting products that are directly related to the defects of the individual powder particles. The consequences of recycling the powder multiple times will be studied in future work. Due to the recycling of the powder, it may happen that the chemical composition of the material used changes gradually. Since the alloying elements (namely, Ti, Ni, Mo) segregate on the surface of the individual powder particles, the chemical homogeneity, which is guaranteed during atomization production, is impaired. An advanced analysis of the mechanism of the creation of new powder particles during the additive manufacturing process is necessary. The particles have bad size distribution and many defects, such as inclusions and layers of segregated alloying elements. For this analysis, a thermal imaging camera will be used to map the particle formation temperatures. Subsequently, thermal analysis methods, such as thermogravimetry, will be used to find the differences between the melting conditions for virgin and reused powders. The melting conditions of reused particles cause defects in the AM builds, which will also be examined.

## Figures and Tables

**Table 1:** The chemical composition of the experimental material, EOS Maraging Steel MS1 [12].

wt [%]	C	Si	Mn	P	S	Cr	Mo	Ni	Co	Ti	Cu	Al	Fe
MS1	≤0.03	≤0.1	≤0.1	≤0.01	≤0.01	≤0.5	4.5–5.2	17.0–19.0	8.5–9.5	0.6–0.8	≤0.5	0.05–0.15	bal.

**Table 2:** Summary table from the measurement of the particle size distribution and circularity for different samples.

Sample	Mean Size [μm]	Circularity
Virgin powder	25	0.93
Reused powder	23	0.91
Residual powder from the sieve	58	0.88
